# Watch who you trust! A structured literature review to build a typology of e-government risks

**DOI:** 10.1007/s10257-022-00573-4

**Published:** 2022-09-28

**Authors:** Bettina Distel, Holger Koelmann, Ralf Plattfaut, Jörg Becker

**Affiliations:** 1 Department for Information Systems, University of Münster, Leonardo-Campus 3, 48149 Münster, Germany; 2 University of Applied Sciences South-Westphalia, Lübecker Ring 2, 59494 Soest, Germany

**Keywords:** Risk, Trust, e-government, Literature review

## Abstract

The information systems, e-business, and e-government literature has unanimously shown that trust and risk are antecedents of the use of information technology and technology-based services. However, a deeper understanding of the relationship between trust and risk, especially when taking into account the extensive knowledge created in fields such as organisational science and psychology, is often missing. With this article, we aim at conceptualizing risk in e-government use. Based on a structured review of the trust-related e-government literature, we derive a typology of relevant e-government risks. We analyse this typology in light of extant trust and risk literature. The typology can be used both to understand the behaviour of system or service users and to design systems and services that can be and are trusted. As such, this research can serve as a basis for future research on the role of trust and risk in designing and using e-government services. The generalizability to e-business services and information systems in general is discussed.

## Introduction

Trust is a major influencing factor for the acceptance and use of information systems (IS) in general (Söllner et al. 2016) and of e-government services in particular (Rana et al. 2015). From a socio-technical perspective, (prospective) users need to trust both the technical component, i.e., the e-government service and the social component, i.e., the service creator and provider. Recent examples of COVID-19-tracing apps show that some citizens do neither trust the tracing app nor the government providing the app (Altmann et al. 2020; Blasimme and Vayena 2020). This ultimately leads to a reduced acceptance of the app.

Trust and risk are two sides of the same coin. Prior research has conceptualised trust in a socio-psychological understanding as the expectations of a trustor, i.e., the one who trusts, regarding the behaviour of a trustee, i.e., the one who is trusted, in situations where the former is not able to monitor, control, or predict the behaviour of the latter (Mayer et al. 1995; Rousseau et al. 1998). As such, trust arises in situations where the trustor perceives risks with regard to the behaviour of the trustee (Das and Teng 2004; Lewis and Weigert 1985).

Risk as a concept is widely used in both IS (Alter and Sherer 2004; Sherer and Alter 2004) and e-government research (Bélanger and Carter 2008; Beldad et al. 2011, 2012b; Horst et al. 2007; Rana et al. 2015). However, it is often conceptualised as a single construct, e.g., IS risks or even more generic simply as ‘perceived risk’. This conceptualisation bears several problems. Firstly, it neglects the perspective that risk may arise from either or both the technical and the social side of the socio-technical system. Secondly, it neglects the multifacetedness of risk that could be shown by prior research (Featherman and Pavlou 2003). Many studies both on e-government and IS in general indicate that perceived risk is not a uniform construct but may, in fact, be differentiated in different perceived risks (e.g., Mendoza-Tello et al. 2019), such as financial (e.g., Soleimani 2022; Susanto and Goodwin 2013) and security risks (e.g., Susanto and Goodwin 2013). Thirdly, it prevents e-government service creators and providers from building systems, which are perceived to be non-risky, i.e., systems that can be trusted. This is especially important as prior research on technology acceptance and adoption suggests that risk and trust influence adoption and use of systems (e.g., Hoehle et al. 2012; Kirs and Bagchi 2012; Seo and Bernsen 2016). As such, a better and more nuanced understanding of risks associated to e-government contributes to a better understanding of trust and, ultimately, a better understanding of acceptance and use of IS and e-government services.

In this article, we aim at providing this increased understanding of perceived risks in acceptance and use of e-government services as one example of socio-technical IS. Specifically, we answer the following research question:

*RQ: How can risks perceived in the context of e-government be typologised?*

Understanding trust and risk as mirror images (Das and Teng 2004), we conduct a structured review of the IS literature (vom Brocke et al. 2009, 2015; Webster and Watson 2002) on trust and e-government acceptance. Here, we expect trust-related research to take into account risk perceptions as well. We analyse over 170 studies and identify risks that have been studied in the context of the acceptance of e-government services before. Next, we use these studies as a text corpus for a qualitative research effort to create a risk typology. We employ a method informed by grounded theory (Charmaz 2006; Corbin and Strauss 2014; Glaser and Strauss 2017; Urquhart and Fernández 2016; Wiesche et al. 2017) and by qualitative content analysis (Mayring 2004, 2015) to collaboratively identify groups and types of risks. Last, we demonstrate the value of our typology through an ex-post outside-in analysis of two real-world examples, the above-mentioned COVID-19 tracing app and the German electronic tax return service.

The remainder of the article is structured as follows. In section two, we present our theoretical background with regard to the relationship between trust and risk. In section three, we discuss our research method. The results of our research are presented in section four. After our demonstration (section five), we close with a discussion (section six) and a short conclusion outlining potentially fruitful avenues for future research in section seven.

## Theoretical background

### On the relationship of trust and risk

Trust research has a long tradition in social sciences and related fields and while up to now no agreed-upon definition of trust exists, consensus has formed around the central dimensions of trust. Trust involves the expectations of one agent, i.e., the trustor, regarding the behaviour of another agent, i.e., the trustee, in situations marked by the former’s inability to (fully) monitor, control or predict the behaviour of the latter (e.g., Mayer et al. 1995; Rousseau et al. 1998). Trust arises in situations that involve a considerable risk or the necessity to take risks for the trustor (e.g., Das and Teng 2004; Lewis and Weigert 1985) and acts as a bypass to these risk perceptions (Öksüz et al. 2016) that make decisions and the corresponding behaviour possible in the first place. Following Rousseau et al. (1998) we understand perceived risks as “the perceived probability of loss, as interpreted by a decision maker” (p. 395), while actual risks are the objective probabilities of such a loss. As such, both trust and perceived risk are concepts based on perceptions held by an individual in contrast to the objectively existing risks and uncertainties of future events (Li 2007).

Although the understanding of risk put forth by Rousseau et al. (1998) is not uncontested, we deliberately apply this socio-psychological concept of trust in this article as opposed to, for example, technical understandings of trust that focus exclusively on aspects of IS security or more economic conceptualisations. Especially in the economics literature but also in sociology, risk has been defined without any connotation of hazard, danger, or negativity. Rather, it is understood as the probable deviation of an outcome from prior expectations (e.g., Tversky and Fox 1995). Jaeger et al. (2001, p. 17, emphasis added), for example, define risks from the perspective of consequences and state that these consequences “[…] are rarely neutral, but carry with them rewards *or* penalties.” In fact, many situations involve outcomes that may either lead to high gains *or* high losses. For a player, a game of roulette is a risky situation. The player makes their bets (e.g., one coin on black, one coin on the number eight). The player here risks losing everything (e.g., the ball lands on a red number), winning back their bet (e.g., the ball lands on the black six), or multiplying their bet (e.g., the ball lands on the eight). As such, from an economic perspective, there is a negative and a positive risk (or a chance). Moreover, the risks associated with this game are objectively measurable and predictable. Nevertheless, we follow the understanding of Rousseau et al. (1998) as it reflects the common understanding of risks as present in IS research (Alter and Sherer 2004; Sherer and Alter 2004) and includes the psychological aspect of *perceived gains and losses*, thus, an individual nuance. More importantly though, this definition accounts for information asymmetries that oftentimes characterise the relationship between trustor and trustee in favour of the latter (Ba and Pavlou 2002; Öksüz et al. 2016; W. Wang and Benbasat 2007). Many interactions, particularly those mediated by technology, come with incomplete information about the interaction partner (W. Wang and Benbasat 2007) and, thus, favour trust as a decision heuristic over the rational calculation of outcome probabilities. In fact, in the situation of a roulette player described above, the player (i.e., the trustor) needs to trust the casino (i.e., the trustee) that the game is not skewed in any way. The game itself does not come with information asymmetries; the player knows the probabilities for the single numbers and combinations of numbers and can bet accordingly.

**Fig. 1:**
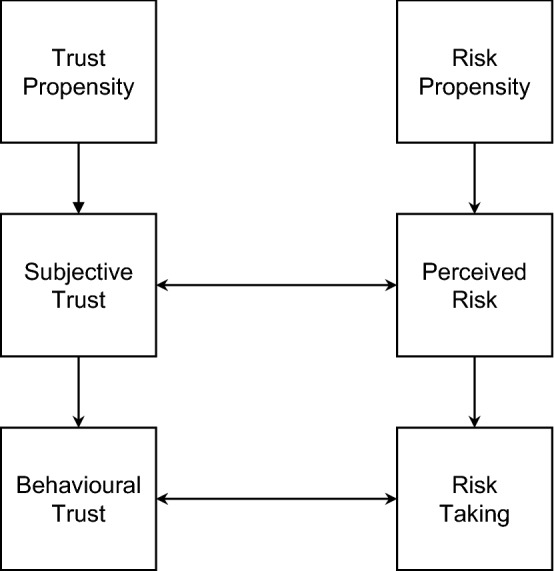
Framework of Trust and Risk. Simplified Adaptation from Das and Teng (2004)

While many conceptualisations of trust directly or indirectly refer to risks (Das and Teng 2004), the relationship of both constructs is still debated. Rousseau et al. (1998), for example, argue that the perception of risks is a necessary condition for trust. Only when an actor perceives risks, trust becomes necessary and may result in risk-taking behaviour (see also Lewis and Weigert 1985). In their seminal work on interorganisational trust, Mayer et al. (1995, p. 726) propose to distinguish trust and trusting behaviours, i.e., taking risks: “Whether or not a specific risk will be taken by the trustor is influenced both by the amount of trust for the trustee and by the perception of risk inherent in the behavior.” In a more recent contribution to this debate, Das and Teng (2004) propose to view trust and risk as “mirror images” (see Fig. 1).

In their view, trust and risk perceptions are subjective probabilities of outcomes (trust as a positive and risk perception as a negative assessment) and depending on their valence, they lead to behavioural trust or risk-taking. Similar to Mayer et al.’s (1995) conceptualisation of trust, Das and Teng (2004) distinguish trust and risk propensity as antecedents to subjective trust and perceived risks, which in turn act as antecedents to the individual’s behaviour (Fig. 1). Irrespective of assumed causalities, these conceptualisations reveal i) the central role of perceived risks for trust and the outcomes of trust, and ii) the subjective nature of both trust and risk perceptions.

### Trust and risk in IS literature

Trust has been recognised as a “sociological reality” (Lewis and Weigert 1985), i.e., a key feature of societies that forms the basis for social relationships. Accordingly, it has been studied in relation to ICT and IS for many years now and in a multitude of different settings such as in virtual teams (e.g., Jarvenpaa et al. 2004; Kanawattanachai and Yoo 2002), in e-commerce settings (e.g., Gefen et al. 2003; McKnight et al. 2002), or in the context of specific technologies such as cloud computing (e.g., Khan and Malluhi 2010; Öksüz 2014). Also, trust is a vital part of the e-government literature (Abu-Shanab and Harb 2019). Lately, particular emphasis has been placed on the question of how and why people trust technologies (Söllner et al. 2016). Consequently, trust research has advanced and can be considered a central topic in IS research as well (Söllner et al. 2018). Broadly speaking, two trust-related IS research streams have emerged, the first focussing on trust between individuals and/or organisations that is mediated through technology, and the second focussing on trust in the technological system itself (Öksüz et al. 2016; Söllner et al. 2012). While scholars have argued conceptually for the very tight relationship between trust and risk, only few studies in both these research streams make this relationship explicit. For example, one of the early articles on trust mediated through technologies by Bélanger et al. (2002) puts emphasis on privacy and security concerns in relation to trustworthiness; yet, the article does not explicate the relationship between trust(worthiness) and risk. In a seminal work on trust in technology, McKnight et al. (2011, 12:4) make the relationship between risk and trust more transparent, arguing that “trust situations feature [the] risk […] that the trustee may not fulfil expected responsibilities, intentionally or not.” Yet, risks related to technology use are neither specified nor measured. Similarly, Söllner et al. (2016, 2018) only mention risks in passing in their works on trust in technology and as a topic of IS research, respectively.

At the same time, risk is a central concept in IS research and has been studied from various perspectives such as IS as a means to manage risks (e.g., Bansal et al. 1993), from the perspective of project or technical risks (see for example an overview by Alter and Sherer 2004; and Sherer and Alter 2004), and from the perspective of users who might perceive or be exposed to risks (e.g., Featherman and Pavlou 2003). As a response to the vast amount of research on this topic, several attempts to categorise risks have emerged in IS research, one of the earliest works being the two-fold publications by Sherer and Alter (2004) and Alter and Sherer (2004). Based on a review of 46 relevant IS articles, they extract three frequently used conceptualisations of risks. *Components of risk* refer to different types of negative outcomes such as financial risks (e.g., loss of money), project risks (e.g., unsuccessful projects), or security risks (e.g., insecure systems). *Risk factors*, in contrast, refer to the sources of risks, such as project management (e.g., tight budgets, size of a project) or actor behaviour (e.g., resistance to change). The third category conceptualises risk as the *probabilities of negative outcomes,* either as “statistical techniques or subjective estimates” (Sherer and Alter 2004, p. 33). As the authors acknowledge, these conceptualisations operate on different levels of abstraction and could easily be divided into further, more fine-grained categories.

A different approach was chosen in a more recent article by Wiesche et al. (2013). Based on a text-mining analysis of the Risks Digest, an online collection of IS risks created by practitioners and researchers, the authors derive a categorisation of IS risks. It consists of ten overarching categories and 30 IS risk clusters. The ten categories include, for example, risks that refer to the use of IS, such as content-related, Internet-related, communication-related, and finance-related risks, or risks that refer to the used infrastructures, such as power supply-related risks and computer-related risks. *E-government risks* is one of the clusters within the category of government-related risks. Hereunder fall risks pertaining to biometric data, voting and elections, and the use of electronic passports. Yet, the categorisation of IS risks proposed in this article is—as a result of the used text mining-approach—a high-level classification of potential risks. In particular, risks pertaining to the use of e-government services are cross-sectional, i.e., they can be related to more than one of the categories identified by Wiesche et al. (2013). Using e-government services comes, for example, not only with risks relating to the government or administrations, but also with financial, computer-related, or Internet-related risks.

A more fine-grained view of risks in the context of e-service use is offered by Featherman and Pavlou (2003) who focus explicitly on the impact of privacy risks on e-service acceptance. The authors propose a categorisation of seven risk perceptions that consumers have to face in e-commerce settings (see Table 1); online purchases are conceptualised as the risk-taking behaviour. Strikingly, all three approaches—by Sherer and Alter (2004), Alter and Sherer (2004), by Wiesche et al. (2013), and by Featherman and Pavlou (2003)—ignore the potentially vital connection of risk and trust. Conversely, works on trust, e.g., those by Söllner et al. (2012, 2016, 2018), mention risks only in passing.

**Table 1:** Types of perceived risks, adapted classification from Featherman and Pavlou (2003)

Perceived risks	Definition
Performance risk	A risk relating to the (under)performance and (mal)functioning of the purchased product
Financial risk	A risk relating to the loss of money through the purchase, secondary costs, and fraud
Time risk	A risk relating to the consumers losing time through the online purchase
Psychological risk	A risk relating to the consumer’s mental state impairing, e.g., resulting from frustration with the online purchase or self-perceptions
Social risk	A risk relating to a changed status of the consumer within their peer group as a result of an online purchase
Privacy risk	A risk relating to the loss of privacy, e.g., by giving away personal information intendedly or unintendedly while making the purchase
Overall risk	An assessment of all other risks taken together

### Trust and risk in e-government acceptance literature

Early on, trust has been identified as a success factor for the acceptance of ICT and IS (e.g., Gefen et al. 2003; Pavlou 2003; van der Heijden et al. 2001). Scholars have argued that acceptance and use of systems may be impeded by risk perceptions that mainly pertain to perceived risks of data security and privacy (e.g., Belanche-Gracia et al. 2015; Milne et al. 2004). Thus, trust has been proposed as a mechanism to bridge these risk perceptions and eventually increase use rates of new systems.

The growing interest of IS scholars on the trust-risk nexus has also informed the study of e-government systems and services (Alzahrani et al. 2017; Belanche et al. 2012; Cabinakova et al. 2013; Carter 2008; Carter et al. 2012). Despite research dating back as far as 2002 (Wang 2002; Warkentin et al. 2002), the field is far from converging towards a unified view of the trust-risk nexus as a recent meta-analysis of studies on citizens’ e-government acceptance indicates (Rana et al. 2015). In many studies, trust is assumed—and in some, shown—to influence the users’ perceived risks (e.g., Bélanger and Carter 2008), yet some studies assume a reversed effect of perceived risks impacting users’ trust (e.g., Horst et al. 2007).

In this article, we argue that these contradicting findings are a result of the oftentimes unclear or even separate treatment of trust and perceived risk. For example, in one of the earlier works on perceived risks and e-government acceptance, Horst et al. (2007) identify two sources of perceived risks, information transmission and information storage and accordingly operationalise risk perceptions with these notions. Also, Bélanger and Carter (2008) differentiate perceived risks into behavioural and environmental uncertainty, the former relating to the unpredictable behaviour of a service provider and the latter pointing towards the Internet as a generally uncontrollable environment. Based on this differentiation, they develop an often-used two-fold understanding of trust (trust in the Internet and trust in the government). Interestingly, the operationalisations of environmental and behavioural risk are rather generic (e-government service use is risky, using e-government over the Internet is risky). What these risk perceptions actually refer to, for example, privacy or security concerns, remains undefined in the empirical assessment. Even though researchers acknowledge the close coupling of trust and perceived risks and even cite risk typologies such as the one by Featherman and Pavlou (2003), many works remain focussed on privacy and security issues, such as the work of Beldad et al. (2012b). Later research focussing on the role of trust of e-government acceptance refrains completely from integrating risk. Belanche et al. (2012), for example, only mention risk in passing as an argumentative reason for the relevance of trust in e-government acceptance.

In an attempt to unify existing e-government acceptance research, Dwivedi et al. (2017) echo the conceptualisation of Bélanger and Carter (2008) by differentiating behavioural and environmental risks. Their operationalisation of perceived risks is, however, more nuanced as they measure perceived risk as one’s concern that personal information might be stolen, as having feelings of uneasiness, as perceiving security and privacy threats, and as the belief to incur negative consequences. They conclude that perceived risk is an e-government specific variable.

These and other examples highlight one peculiarity we aim to address with our research: While IS research in general has established various classifications of IS-related risks (Alter and Sherer 2004; Featherman and Pavlou 2003; Sherer and Alter 2004; Wiesche et al. 2013), e-government acceptance research is, in this regard, rather fragmented and often-times uses vague or very general operationalisations of perceived risks. The used conceptualisations and operationalisations are often-times focussed on privacy and security issues, whereas other probably important dimensions as highlighted in the discussed typologies and classifications (e.g., social risks, psychological risks, technology-related risks) play a minor role. More importantly though, many works are built on knowledge from the e-commerce domain; a comprehensive view on perceived risks that are specific to the use of public e-services as suggested by Dwivedi et al. (2017) is yet missing.

Against this background, our work aims at providing a more nuanced view of perceived risks and their relation to subjective trust by deriving a typology of perceived risks in e-government use.

## Method

### Data collection

**Fig. 2:**
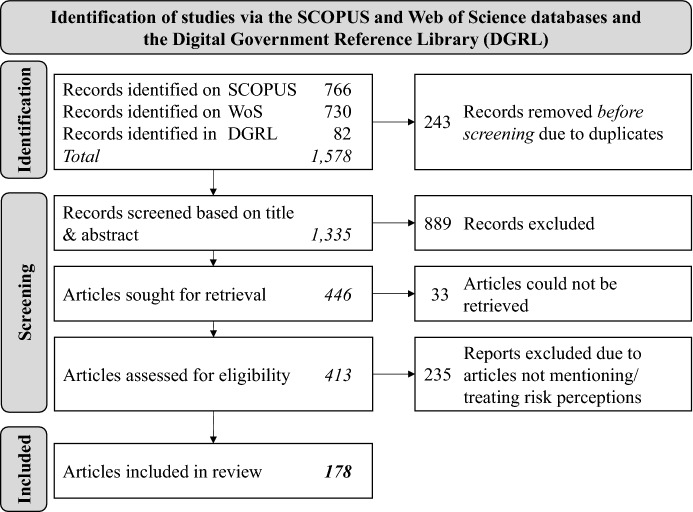
Data collection process (depiction based on Page et al. 2021)

Many scholars have devoted publications to the trust-acceptance nexus and, consequently, there is a large body of knowledge to consider for this study. As such, we opted for a structured literature review approach (vom Brocke et al. 2009; vom Brocke et al. 2015; Webster and Watson 2002). While our initial search in the conceptualization phase (vom Brocke et al. 2009) was focused on recent articles in leading journals in the information systems field (as suggested as a starting point by, for example, Webster and Watson 2002), we later expanded our search to be reasonably broad. The body of literature used in this study was collected until January 2022. In order to conceptualise a typology of risks in trust-related e-government literature, we first searched the databases SCOPUS and Web of Science as well as the Digital Government Reference Library (DGRL; version 17.5), using combinations of search terms such as e-government, trust, and acceptance.^1^ As such, we combined results from two general databases with one topic-related database to ensure near-full coverage of the topic. Building on this very open and broad search and considering that we did not use any restrictions regarding publication year, we deemed an additional forward and backward search to be not necessary. This search led to 766 articles from SCOPUS, 730 articles from Web of Science, and 82 articles in the DGRL. After combining results from all databases and deleting duplicates, we further excluded non-English sources and news articles, resulting in 1,335 potentially relevant distinct articles. Based on the titles and abstracts, we reduced the sample to 446 articles that explicitly dealt with citizens’ trust in and acceptance of e-government. Of these articles, we could not access 33 entries despite using the access of various universities. Only now, we considered the full texts and searched them for sections dealing with risks in the context of e-government use, leaving 178 articles in the final sample. The data collection process is depicted in detail in Fig. 2.

### Data analysis

In order to extract the different types of perceived risks addressed in relation to trust and e-government acceptance, we used directed qualitative content analysis with elements of inductive category building following suggestions by Hsieh and Shannon (2005) and Mayring (2015). The chosen approach is comparable to data analysis techniques used in grounded theory studies (e.g., Glaser and Strauss 2017; Urquhart and Fernández 2016; Wiesche et al. 2017) and consists of the following steps: highlighting text (Hsieh and Shannon 2005), coding highlighted text with predefined categories (Hsieh and Shannon 2005; Mayring 2015), refining existing and adding new categories (Hsieh and Shannon 2005; Mayring 2015), and finally, working through the text corpus (Mayring 2015). For the second and third step, we used constant comparisons and memoing (Wiesche et al. 2017) as techniques to enable categorisation of text elements.

*First,* we extracted all statements from the articles in which perceived risks were mentioned. We used the documents search functions for this procedure, searched for ‘risk’, and then manually copied the sentences containing the search word as well as adjacent sentences that gave context to the corresponding statement in the MAXQDA software commonly used for qualitative analyses.

*Secondly,* we used the first thirty statements, ten per researcher,^2^ as a starting base, individually read through these statements, and extracted first categories for the perceived risks. We focussed solely on those risks perceived by citizens in order to keep the complexity of our typology manageable. Additionally, our analysis revealed that most research on trust and e-government acceptance takes this perspective, too, and other perspectives (administration employees, business users) were severely underrepresented.

As initial coding scheme, the categories of perceived risks by Featherman and Pavlou (2003) were used. Statements that fitted with the categories’ description as put forth by Featherman and Pavlou (2003) were assigned to their categories. Additionally, we coded statements from the articles that were either conflicting with the initial categories or indicated a need to refine the categories by Featherman and Pavlou (2003). All statements referring to perceived risks that could not be assigned to one of their categories were coded as potentially new categories and memos containing a first generalised description of these potentially new categories were added. This process was already iterative in that each of the researchers frequently went back and forth in their assigned batch of articles to refine categories or apply categories developed from later articles on those already analysed (constant comparison). After this initial analysis, we discussed our findings to consolidate and refine the emerging categories across the three samples. As such, the initial categories can be understood as a first instance of open codes that emerged from the data (Wiesche et al. 2017).

Initially, we focussed this step on the *types of risks* mentioned in the articles and tried to extract the meaning of each mentioned risk type. For example, many papers refer to performance risks (e.g., Kollmann et al. 2015; Rotchanakitumnuai 2009) and initially these statements were all assigned to the corresponding category by Featherman and Pavlou (2003). Yet, a closer examination shows that either this type of risk is not explained at all (e.g., Alzahrani et al. 2018) or we came across very different understandings subsumed under the same label (e.g., Ahmad et al. 2011 vs. Kollmann et al. 2015 and Rotchanakitumnuai 2009). Thus, in the first round of coding, we already highlighted these critical statements and used memos and preliminary categories to collect more instances of similar cases. However, after reading only a handful of the statements we realised that there was more conceptual vagueness to disentangle. Subsumed under the label ‘perceived risk’, for example, and in addition to different *types of risks*, many researchers also addressed *consequences* of this risk perception for individual actors (e.g., Kollmann et al. 2015) as well as *sources* from which these risks might stem (e.g., Papadopoulou et al. 2010). Consequently, we decided to analyse these categories, too, and used them as a first-level differentiation; categories such as time or performance risks were used as second-level categories. This can be understood as axial coding (Wiesche et al. 2017) that employed both inductive and deductive thinking. For example, under *sources of risk perception*, we listed different aspects such as *infrastructure/technology*—further specified into *own device* and *Internet*—or *third party.* In the initial step, these categories and their specifications were kept as close as possible to the original wording in order to not impose meaning on the text elements.

The resulting first draft of categories contained the original categories by Featherman and Pavlou (2003), but also introduced new first-level categories (types of risk, sources of risks, consequences of risk perceptions) and new second-level categories (e.g., control of service). This draft was then applied to the next set of 10 statements per researcher. The above-described process was repeated, only this time, we developed first definitions for the new categories and their specifications to ensure a coherent understanding among the researchers. This iteration was ended with a re-crafted draft of categories.

*Thirdly,* we assigned one third of all statements, including the ones from the preceding steps, to each researcher and used the drafted coding scheme to code the first half of the statements. After this round of coding, the results were again discussed, the coding scheme refined and consolidated and re-applied to the material, this time to all statements.

Changes occurring in the course of this round were again discussed and applied to the coding scheme, and—where necessary—codings of the material adapted. Since the changes after this iteration were only marginal, we decided to move on to the next and final step and compare our understanding of the coding scheme in a more formal way.

Thus *fourthly*, we applied a check of intercoder agreement to ensure that our understanding of the coding scheme was coherent. For this, we re-assigned the sets of statements among the three researchers and completely re-coded one set with the coding scheme from step three. The resulting codings were compared and discrepancies discussed. In this step, no further categories emerged from the material, although we adapted some of the initial category definitions and consolidated the categories, i.e., too fine-grained categories were merged. The coding scheme resulting from our process of data analysis is presented as a typology of perceived risks in e-government acceptance and shown in Table 2. For each category, we include representative quotes from the literature reviewed.

**Table 2:** Typology of perceived risks in e-government acceptance

Description	Representative quotes
**Where does the risk stem from?** Provider *System/Service*	
Provider refers to the entity offering either the service or the system to its user. It is the entity liable for the provided service or content and may be congruent with the developer. Also, system and service provider may be the same entity. In the context of e-government, the provider is commonly the administration, the government, or any other public agency, but can also be a private company commissioned by the government, e.g., a private IT service provider or system integrator	“Moreover, every governmental institution resembles a monopolistic ‘business’ entity that provides services exclusive to a country […]. Without exposure to market forces, governmental institutions are often laden with a supplementary layer of political affinity. With e-governments acting as surrogates (or proxies) for governmental institutions, citizens may be compelled to question the aspirations and motivations behind such systems […]” (Lim et al. 2012, p. 1112) “Perceived risk is more related to the security of the government’s databases” (Roy et al. 2015, p. 358)
Developer *System/Service*	
Developer refers to the entity responsible for the technical development of a system or service. Causes for risk perceptions included in this category may refer to negligence but also to malevolence	“A national information infrastructure is a sociotechnological network of people (stakeholders), hardware (networked systems), software, and security and privacy policies that must deal with risks (threats such as equipment failure, extreme weather, hacking, and sabotage)” (Hole 2016, p. 69) “Distrust in e-voting systems and, as a result, low electoral activity of citizens may also arise due to lack of trust in developers and vendors who could provide the equipment and software solutions in the area.” (Kassen 2020, p. 321)
Third Party	
Third Party refers to, for example, hackers. It includes only parties that are not an intended party in the interaction of user and provider and developer	“Personal information shared with an organization digitally could either be exploited by the organization collecting the information or by unauthorized third parties that could access such information using sophisticated technologies.” (Beldad et al. 2012a, pp. 41–42)
Infrastructure/Technology *Internet/Own Device*	
Infrastructure may comprise the Internet in general as a source for risks, but also less generic technologies such as cloud computing or even the users’ own device	“In effect, negative experiences with the internet tend to increase concerns about internet risk, leading to a decrease in trust in online services.” (Alzahrani et al. 2018, p. 141)
Users *Administration (Employees)/Businesses/Citizens*	
Users are the human entities or groups of human entities that are offered a service/system or supervise a service (administration employees). Risks can stem from users’ inabilities to handle technology, their lack of knowledge or unwillingness. For the sub-category administration employees, we further consider statements that refer to the (malicious) behaviour of individuals within an organisation, particularly when this behaviour is in contrast to the general behavioural norms of the institution. For example, risks may arise through the misuse of personal data by government staff, although this behaviour is not tolerated/encouraged by the government	“Some individuals may also view the political world as corrupt and deceitful. Some voters may fear that political elites could somehow sabotage an online vote in their favor; […]” (Powell et al. 2012, p. 363)
Act of God/ Environment/ Emergency	
Risks can also arise from circumstances outside the control of the individual or an organisation, for example in case of natural catastrophes	“The second type of uncertainty is environmental, which originates because emergencies, by their nature, cannot usually be predicted in their exact timing or severity.” (Aloudat et al. 2014, p. 155)
**Who perceives a risk?**	
*Internal entities*	
Employees may perceive risks in the use and provision of e-services	–
*External entities*	
External entities can be businesses other organisations, and citizens. Citizens as users of e-government services can perceive various risks that may or may not be congruent with actually existing risks	“In the context of e-government, perceived risk can be seen as the conviction by a citizen that he/she will suffer some sort of loss when using an e-government system.” (Verkijika & Wet 2018, p. 85) “In face of risky situations, decision makers, i.e. citizens, need trust as a bypass to these risk perceptions to be able to decide and act.” (Distel et al. 2021, 164)
**What is the type of risk?**	
Quality of information/data *System-provided/User-driven*	
Quality of information/data pertains to, for example, accuracy, completeness and timeliness of data (according to Ballou & Pazer 1985; 1995). This might relate to both information *provided to citizens* and information on citizens *stored and processed by administrative* bodies. Furthermore, risks might occur on the part of users as they have to provide their information timely, accurately, and complete as well	“The citizens’ perspective—The factors for acceptance include familiarity or experience with e-services and government; ease of use; perceived usefulness; trust in the organisation and service for example interacting with government on-line and the perceived safety/risk of providing information to government; perceived quality of information and service; and perceived behavioural control and subjective norms […]” (Tassabehji & Elliman 2006, p. 3) “I believe the information offered by the m-government applications is genuine […] I can rely on m-government applications for information about different services.” (Eid et al. 2021, p. 471)
(Information) Security *Confidentiality of information, data/Integrity of information, data/Availability of information, data*	
This category subsumes all statements that refer to the technical and information security of e-government systems and services as a potential risk *Confidentiality* refers to the risk perceptions that unauthorised parties get access to personal (or in rare cases: governmental) information. This includes any form of privacy risks *Integrity* refers to the risk perception that information received, provided and/or stored by organisations is incomplete, has been changed, or is inconsistent. Integrity refers to the immutability of information/data *Availability* refers to the risk perception that information might not be available to the service/provider/user. This category may include risks pertaining to the (technical) reliability of services. Hereunder fall also statements that indicate that a system or a service is not or not continuously available	“This implies that third parties can intercept, read and modify the information.” (Horst et al. 2007, p. 1839) “Although cloud computing can benefit e-government services, there are risks, both tangible (access, availability, infrastructure, and integrity) and intangible (reliability of the cloud, security, safety mechanisms, data confidentiality and privacy, and so on).” (Lian 2015, p. 100) “SEC1: Hackers may be able to intrude into government websites and steal my personal information stored on the web. SEC2: I would not feel secure sending sensitive information to e-government websites. SEC3: Overall, it is not safe sending sensitive information to e-government websites.” (Alzahrani et al. 2018, p. 132)
What is the type of loss?	
Asset-based losses	
*Personal data*	
Hereunder fall statements that refer to information of citizens that is actually lost in the process of service provision and cannot be restored	“In addition, e-government transaction risk can also involve loss of data which are of high importance to the citizen, beyond typical privacy concerns faced in e-commerce, such as tax or health information.” (Papadopoulou et al. 2010, p. 4)
*Financial resources*	
Statements of this kind indicate any form of financial loss for the service users. This might refer to payments that need to be made in order to receive a service, financial investments, or missed opportunities for financial gains	“Financial risk accounts for the potential monetary outlay associated with purchasing or maintaining a product or service […]. This concept includes criminal activity such as fraud.” (Kollmann et al. 2015, p. 309) “A group of citizens raised a concern regarding the use of their internet banking credentials for authenticating in these digital public services, as they believed that revealing their internet banking credentials might pose risks of their misuse by government (e.g. for taxation or other purposes); this reveals an important mistrust in the government concerning the way of use of this banking-related data provided by the citizens, which affects negatively the attitude of citizens towards the use of these e-services.” (Loukadounou et al. 2020, p. 231)
*Time*	
Statements indicate that actors can lose time or that the given process/behaviour might require more time as compared to another (traditional, habitual, status quo) process/behaviour	“Time risk is associated with the loss of time an individual may suffer owing to wasting time, for example, on opaque offers or useless sites […]” (Kollmann et al. 2015, pp. 309–310) “TR1: Using e-government websites to search for or request a government service could take up my time. […] TR2: Using e-government websites to search for or request a government service will require a lot of time TR3: Using e-government services will not waste my time.” (Alzahrani et al. 2018, p. 132)
Interpersonal losses	
*Social risk*	
Hereunder fall risks pertaining to the social status of a person, their reputation and/or standing in the peer groups. It may also relate to the (anticipated) judgement of one’s behaviour by peers	“Social risk refers to the potential loss of status in a social group resulting from adopting a particular product or service […]” (Kollmann et al. 2015, p. 310) “In addition, social risks can occur as people might fear social pressure or social exclusion from using or not using the tracing app.” (Oldeweme et al. 2021, 2)
*Control over product/service* Loss of democratic rights	
Statements indicate that the actors lose control over the service consumed or product purchased. This category contains only statements that refer to the product/service, not statements referring to the availability of a system/information/data (see ‘availability’) A special form of lost control may be the loss of democratic rights that may occur in the course of electronic voting, for example	“First, task uncertainty and workflow uncertainty arise from the service process of e-government. When using an e-government service (e.g., online tax filing), citizens need to be provided with necessary information (e.g., user instructions and status updates) to accomplish service tasks (e.g., filing taxes) and keep track of the service workflow (e.g., checking tax refund status). With incomplete information, citizens may feel uncertain about how they can obtain desired services, and when and which government agencies will receive and process their service requests.” (Venkatesh et al. 2016, p. 90)
Intrapersonal losses	
*Physical health*	
This category subsumes all statements that indicate that an actor risks his or her physical health in the course of requesting a service	“First, a perception of a personal risk could originate when the user is uncertain whether or not the LBS [location-based service] infrastructure would cope with the emergency situation, which might lead to a potential risk to the personal safety or the safety of important others (i.e. family members, friends, or working companions).” (Aloudat et al. 2014, p. 155)
*Psychological/mental health*	
This category subsumes all statements that indicate that an actor risks his or her mental health in the course of requesting a service. This includes statements referring to a general uneasiness related to e-service use, but also more severe forms of mental health issues such as fears	“Psychological risk occurs when an individual’s selection or performance has a negative impact on the individual’s peace of mind or self-perception […]. This includes feelings of frustration at not being able to achieve the expected outcome of a process or action […]” (Kollmann et al. 2015, p. 310)

*Lastly*, we demonstrated the value of the resulting typology through an application against a recently introduced e-government service, i.e., the German Covid-19 tracing app, and against a long-existing e-government service, i.e., the German electronic tax return service. To this end, we familiarized ourselves with both services through personal usage and a review of published media articles. Based on this, we identify several risks (potential) users might perceive. This evaluation through demonstration is informed by prior research on the design science methodology (Peffers et al. 2006; Sonnenberg and vom Brocke 2011, 2012).

## Results: typology of perceived risks

We extracted the different types of risk perceptions mentioned in trust-related e-government literature. While some articles include definitions of the term ‘risk’ or ‘perceived risk’ that refer to a more general understanding of the term, we shed light onto the specific risks or instantiations of these understandings. For example, Schaupp and Carter (2010) provide a general understanding of what a user’s risk is (a belief that the individual may incur losses) and later on specify this understanding with examples, such as an online vendor taking advantage of the user. This analysis, thus, includes any specific risk mentioned by the authors and is not limited to either theoretical or empirical sections of the studied articles. Moreover, it needs to be noted that the perception of these risks might depend on the existence (or non-existence) of trust as conceptualized in Sect. 2.1.

Overall, we extracted four overarching categories and 17 sub-categories (see Table 2). The **four general categories** differentiate between the thing or entity causing a risk perception (*Where does the risk stem from?*) and the entity experiencing this risk perception (*Who perceives a risk?*). These two categories mirror the distinction of a trustee (the party receiving trust) and the trustor (the party trusting) present in trust research, a distinction so far not explicitly made in e-government research. While generally business users and administration employees could also perceive risks in relation to e-government services, these perspectives are out of scope in this article. We then identified different types of risk perceptions (*What is the type of risk?*), i.e., the entities to which risk perceptions refer, which are information and data. Again, this category is not included explicitly in conceptualisations of perceived risks, which divide the nature of perception into, for example, expectations or feelings (e.g., Kollmann et al. 2015), but do not specify what the subject of this perception is. The qualitative analysis of extant e-government acceptance literature also includes statements that describe the types of losses that can occur if the perceived risk materialises (*What is the type of loss?*) such as financial losses.

Within each of these general categories, we identified several *sub-categories* that are the content-related manifestations of the superordinate categories. For example, the types of risks are divided into ‘quality of information/data’ and ‘(information) security’, where quality pertains to the accuracy, timeliness, and completeness of received, provided and stored information/data (Ballou and Pazer 1985, 1995; Lee et al. 2002), and where information security pertains to the technical and information security of an e-government system or service (Perrin 2008; Salisbury et al. 2015). As these sub-categories are still rather broad—at least in some cases—we extracted further *specifications* where needed. For example, we identified users (sub-category) as a potential cause for risk perceptions (general category), but saw a need to further specify this category as either referring to businesses, administration employees, or citizens as users of an e-government service or system (specification).

During the last round of coding (see above), three groups of losses (fourth category, see Table 2) emerged from the data. *Asset-based losses* are all losses pertaining to one’s possessions, material and immaterial. While losing money through the use of e-government services is an example for a material asset-based loss, the destruction of personal data is an example for an immaterial asset-based loss. *Interpersonal losses* are all losses arising in relation to or affecting one’s relation with their environment. Social status and reputational damages are interpersonal losses that affect the users’ relationship to their environment, while the loss of control over a product or service can only arise in the interaction of the individual with its environment. Finally, *intrapersonal losses* refer to jeopardising or losing one’s physical and psychological integrity. This might either refer to the user’s mental or physical health.

Interestingly, many conceptualisations also refer to the consequences of perceived risks for the users’ behaviour, although scholars as Mayer et al. (1995) and Das and Teng (2004) argue for treating perceptions and actions separately, i.e., risk perceptions and risk-taking behaviour and trust and trusting behaviour are conceptually separated (see also Fig. 1). Yet, many articles in our sample are not as clear-cut as this and mingle the risk perceptions with its consequences, such as increases or decreases in trust, changes in use intention and behaviour, and perceptions of the service’s usefulness.

## Demonstration of typology using two thought-experiments

In this section, we present two examples of how the typology can be used to identify risk issues related to an e-government service. We conduct thought experiments based on publicly available secondary data (mainly news coverage) using two contrasting cases to evaluate the applicability of our research result (inspired by the evaluation pattern *demonstration* as introduced by Sonnenberg and vom Brocke, 2011; 2012). The first example, the German Covid-19 tracing app, serves as an example for a public service that has been developed and rolled-out under extreme circumstances. This case is complemented by our second example, the electronic tax return, a well-established public e-service and the most often used one throughout Germany.

### First evaluation: COVID-19 tracing app

With the advent of the ongoing COVID-19 pandemic in early 2020, the urgent need to trace infections and warn potential patients resulted in the development of Corona tracing apps. Since then, many countries have launched apps that enable users to trace contacts with potential COVID-patients (Johnson 2020; O'Neill et al. 2020). Prior research could show that the use of COVID-19 tracing apps is heavily influenced by perceived risks (Amann et al. 2021; Lin et al. 2021; Reith et al. 2021). Exemplarily, Munzert et al. (2021) could show that people living in high risk-incidence regions or having COVID-19 cases in their personal network were more likely to use a COVID-19 tracing app. They also identified that a strong perception of COVID-19 as a threat to oneself or one’s friends as well as trust in the government, science, or the healthcare system were drivers of app use (Munzert et al. 2021). Applying the developed typology to this type of tracing apps allows us to demonstrate the added value of the typology in an extraordinary, yet illustrative setting.

Hit by the pandemic with an unforeseen force, public administrations around the world were challenged to provide easy-to-use, secure digital solutions for contact tracing and informative purposes and had to do so fast. The public demand and uptake was similarly unforeseeable: When the Federal Government of Germany released its CoronaWarn App in June 2020, it was met with great trust from the citizens and praised by non-governmental organisations for being open source and for its high data security standards (Kutschera 2021; Simon and Rieder 2021). Within only two weeks, it had been downloaded 15 million times, but already in August 2020, use intention was decreasing considerably, while the expectation that the app use would not impact anything increased (Scheiber et al. 2020). Pressured by recent developments and competing apps developed by private firms, the app today also enables the registration for events and the storage of vaccination certificates. Businesses can use the app for their events or stores by providing event attendees, sports club members, or customers in stores and restaurants the option to anonymously checking in (via QR codes) and being informed or informing others about a positive COVID-19 test (Deutsche Telekom AG and SAP SE 2022). Using this example of a public e-service, we highlight the applicability of our typology as a tool to analyse potential and existing issues with an e-government service.

**Sources of risk perceptions** Commissioned and eventually *provided* by the Federal Government, the CoronaWarn App is *developed* by SAP and Deutsche Telekom. While the latter “is providing the network and mobile technology and will operate and run the backend […,] SAP is responsible for the app development, its framework and the underlying platform” (Deutsche Telekom AG and SAP SE 2022). Additionally, developer teams from both companies are supported by the open source-community. Thus, risk perceptions may be caused by these four entities. While many studies in the field refer to administrations—and to a lesser extent administration employees—as the main interaction partners, our typology is more fine-grained as it includes further relevant entities such as system developers and providers. In the chosen case, the developers are of particular interest as they have been covered by media quite extensively and we can, thus, assume that users are fully aware of the app being developed by Deutsche Telekom/SAP but provided by the Federal Government.

The app heavily relies on the inputs from the community of *users*, i.e., citizens need to not only activate the app on their mobile phones but need also to register positive Corona tests, use the QR codes to register for events, and store their vaccination certificates using the app. Malicious but also unintentionally wrong behaviour may create risk perceptions for other users. While the risk of hacking is comparably low as data is not centrally stored on servers, risk perceptions may relate to the security of one’s own device which is used to store personal data such as the vaccination certificate.

**Risk perceivers** Obviously, *citizens* using the app may perceive risks, ranging from risk perceptions related to the provider and developers of the app over other users to the used infrastructure and their own devices. However, with the recent updates and developments, also business users may perceive risks when using the app as an event management tool, for example. Their risk perceptions may also refer to the provider and developer, but also to the community of users as the security of their event or business heavily relies on the app users’ behaviour.

**Types of risk perceptions** *The quality of information provided to citizens* is crucial as the app includes current incidence rates (7 days), the number of confirmed new infections, the number of persons using the app to warn others (i.e., the number of persons who have registered a positive COVID-test), the rate of hospitalizations, and the current R-value. Furthermore, the app includes as its core feature the user’s risk status, i.e., it traces whether the user has been in contact with another person tested positive for COVID-19. Consequently, the app not only relies on information provided by public agencies, but also on the *quality of information provided by the users* and does so more than probably most e-government services. If users who tested positive for COVID-19 do not include this information via the app (on time) or refrain from registering for events using the app, others may not be warned. Again, we can assume that app users are fully aware of the relevance correct and complete data have for the overall success of the app as the app has been discussed extensively by media (Amann et al. 2021) and has been promoted as a success factor by the Federal Government and other public agencies.

Since information on positive tests and event registrations is only stored on the users’ mobile phones and is not stored centrally, *information on citizens* may be of lesser importance in this particular case.

As with any digital service, app users’ may perceive risks of *confidentiality* if unauthorised parties gain access to their personal data stored with the app (positive tests, vaccination certificate, event registration, contact diary). In this regard, especially when first plans of the CoronaWarn app appeared in the media, the possibility to track the location of users was heavily discussed as a potential risk. However, ultimately it was opted to not track the location of users but, instead, to employ the anonymous IDs of users in range of the Bluetooth module of the smartphone (Blom et al. 2021; Kerkmann and Scheuer 2020; Robert Koch-Institute 2022). Additionally, *integrity* and *availability of information* may become issues if users (businesses, citizens) or the provider (government) fail to make all information available to the users or if the information provided is inconsistent.

**Types of losses** In contrast to the preceding categories, not all types of losses are important in our chosen example. The app can store vaccination certificates and event registrations, thus, users may perceive the risk that this *personal information can be lost or destroyed* due to the app’s malfunctioning or interventions by third parties. However, this risk can be regarded as being marginal as vaccination status is mainly stored on paper. Citizens may even fear *losing democratic rights* if they do not use the app or if they register a positive test, for example, because they might be excluded from events, not admitted to stores, or not even allowed into public offices or electoral offices. Finally, they may perceive risks to their *psychological or physical health*, for example, resulting from not being sufficiently informed and warned about contacts with infected persons. Again, whether these losses can actually materialise or not is not so much of question as is *the individual’s perception that these losses can occur*.

### Second evaluation: tax return service

In 1996, the German federal government and the 16 state governments decided to introduce an electronic tax return service (Elektronische Steuererklärung, electronic tax declaration, ELSTER). Available to tax payers since 1999, the service soon became a frequently used tool for business users, with about 90% of all companies filing their taxes online in 2005 (Krebs 2005). Since 2012, ELSTER became mandatory for all individuals who have business-related income (e.g., freelancers, small business owners) (EStG 2009). While citizens’ adoption started off slower with only 20% of the individual tax payers using ELSTER in 2005 (Krebs 2005), the service is today the largest and best-known e-government application in Germany with over 31 million income tax statements having been filed in 2021(Bayerisches Landesamt für Steuern [Bavarian State Office for Taxes] 2022), which makes up around 75% of all tax payers in Germany. The development and maintenance of ELSTER is coordinated by the Bavarian government, more specifically the Bavarian State Office for Taxes (Krebs 2005).

**Sources of risk perceptions** Risks with regards to ELSTER can stem from the behaviour of the government (as a *provider* and *developer*). Early on, German media speculated that ELSTER could be one way for governmental agencies to install spyware as a means of gaining insights into potentially criminal behaviour of citizens (Hoyer 2007). Following this line of argumentation, at least one company went to court to argue against the mandatory use of ELSTER. The company argued that installation of government-provided software bears high risks which outweigh the advantages of electronic tax filing (Finance Court of the City State Bremen [Finanzgericht Bremen] 2014).^3^ Moreover, risks can also stem from *third parties*. In the beginning, filing tax returns was in parts possible just with a tax identification number. As such, third parties could file tax returns for other businesses. Moreover, risks could also originate in the underlying *infrastructure* (Akkaya et al. 2013). Due to network outages, businesses and citizens might miss deadlines of filing their taxes. Lastly, risks also stem from the *users* themselves. Media highlighted the need to file the taxes correctly—errors are apparently more severe when using ELSTER than when using traditional paper based tax return processes (Hoyer 2007). Additionally, experts claim that the personal computers used for tax filing are oftentimes insufficiently protected (Hoyer 2007).

**Risk perceivers and types of risk perceptions** As already introduced above, risks with regards to the use of ELSTER can be (and are) perceived by both *private citizens* and *businesses*. They both perceive risks with regards to *confidentiality* of information and *integrity* of data.

**Types of losses** From the use of electronic tax return services such as ELSTER, we can mainly derive two potential losses. Firstly, individuals and businesses might see the risk of *financial losses*. This relates both to potentially too high tax payments and to risks pertaining to the payment information that is used in the digital tax return service processes. Secondly, filing taxes electronically may also lead to *time losses* as compared to filing taxes on paper or hiring a tax advisor. German tax payers need—irrespective of the medium—a considerable amount of time to prepare their taxes, on average 10 h (Blaufus et al. 2019). Thus, a loss could also be perceived if citizens or businesses use ELSTER and spend considerable time filing the taxes on their own as compared to, for example, hiring a tax advisor who could file taxes on their behalf. Losses may arise from learning how to use the software and using the abundance of tax-related information provided by the software (Blaufus et al. 2019). Thirdly, especially individuals might also see *social risks*. Here, the potential publication of private data on amounts of income, tax deductible donations to political parties, or owned properties might lead to changed social status, i.e., social risks.

## Discussion

### Typology of risk perceptions: discussion

This article sets out to find answers to the questions *How can risks perceived in the context of e-government be typologised?* In answer to this question, our analysis reveals that perceived risk in trust-related e-government acceptance literature is discussed with many different notions; the field is far from converging to an agreed-upon understanding of and, consequently, operationalisation of risk perceptions. Structuring the different views on risk perceptions leads us to propose the typology presented in Sect. 4; it is the first attempt to systematically unravel the oftentimes unclear and even contradicting meanings of ‘perceived risks’. Our qualitative analysis of 178 research articles of the e-government domain leads to the extraction of four overarching categories that are sub-divided in 13 sub-categories. The overarching categories differentiate the cause of risk perceptions, the entities perceiving a risk when faced with e-government services, the types of risks, and the types of potential losses as perceived by the users.

Herein lies our *first contribution* to current e-government research. This typology enables the systematic study of how risk perceptions emerge and to what objects of the overall e-government system they refer to, i.e., to the social system (providers, developers, users) and/or to the technical system (devices, infrastructure). Moreover, the typology includes the types of risks prevalent in e-government service acceptance. The differentiation of risk types in quality-related risk perceptions and the confidentiality, integrity, and availability of information or data highlights a need to overcome the commonly-used, two-fold conceptualisation of perceived risk in privacy and security risks (Belanche-Gracia et al. 2015; Beldad et al. 2010, 2011, 2012b). Again, the proposed typology is more fine-grained and helps in identifying what security and privacy are actually related to. More than that, the quality of information as a risk perception-type adds to this differentiation. Then, we also identified losses citizens face and differentiate these in asset-based, interpersonal, and intrapersonal losses. Arguably, facing financial losses may have a quite different impact on one’s assessment of an e-government service than facing health risks. In addition, some of the risks are long-term in their consequences (e.g., health issues, financial losses), while others can be overcome through habituation (e.g., time losses). This benefit could be shown also in the demonstration with the case of the COVID-19 tracing app.

Our *second contribution* lies in the relational view of perceived risks that we provide with the proposed typology. The explicit differentiation between an entity causing and another entity perceiving a risk, is—to the best of our knowledge—novel as no other research makes such a clear distinction in the IS and the e-government field. Commonly, most studies on e-government acceptance have strong focus on the risk-taker and are less clear on where the risk perceptions stem from (e.g., Bélanger and Carter 2008; Choi and Song 2020). However, to differentiate sources of risk perceptions from the risk-taker enables us not only to better analyse and understand the type of risks and losses involved in each relationship, but also to conceptualise trust as a mirror image of risk (see following section).

### Relation of risk perceptions and subjective trust in e-government acceptance literature

The relational view on perceived risk as proposed by our typology enables a more nuanced perspective on trust as well. Thus, our *third contribution* lies in the consequences of differentiating different sources of risk perceptions for the corresponding subjective trust of users. If risk perceptions stem from either the social or the technical system and are perceived by the users (risk-takers), the latter are also the trustors placing their trust in the technical or social system. While implicitly prior research suggests a corresponding relationship (e.g., Bélanger and Carter 2008) by differentiating trust in the Internet and trust in the government, the typology proposed in this article makes these relationships explicit and is more fine-grained as the social system in particular is pictured in greater detail and not only refers to administrations or governments in general but to different entities (provider, developer, system, service). While much previous research uses trust in the Internet and trust in the government as central concepts, other trustor-trustee-relationships are not systematically considered (e.g., trust in the service provider, trust in the system provider, trust in developers). Thus, our typology suggests that the existing trust relationships in the context of e-government are far more complex than considered so far. Although scholars have acknowledged that “[…] e-services are complex, social-technological systems, comprised of multiple elements that could invoke distinct trust beliefs” (Belanche et al. 2014, p. 627), a systematic and nuanced view on the different sources of risk perceptions—and consequently subjective trust—as well as their consequences has not been offered so far. The differentiation of various risk sources as proposed by our typology is even more important as scholars suggest a trust transfer-effect for citizens’ use of e-government services, where citizens’ trust in the public administrations and the used technology acts as a proxy for trust in the public e-service (Belanche et al. 2014). Future research, thus, needs to investigate whether the different risk perceptions included in our typology add to the effect of trust transfer. This seems even more important considering that current research does not yet consider the role of non-governmental actors in the context of citizens’ use of and trust in public e-services (Rana et al. 2015).

In our typology, we differentiate several types of losses; however, not all losses may be equally important. It is reasonable to argue that time losses are valued lesser by citizens than potential losses of money or personal data, for example. Consequently, being faced with the potential loss of highly valued assets may require a solid trust basis to cope with these risk perceptions, whereas other losses may not require trust at all. Again, these nuances, though discussed in literature, are not reflected in empirical investigations on that matter (Rana et al. 2015), where perceived risk is treated as a unidimensional/uniform construct or where different types of risk perceptions are considered equally (Featherman and Pavlou 2003). We conclude that depending on the salience of different risk perceptions the level of trust needed to cope with these risks differs. The overall notion of current works that subjective trust or perceived trustworthiness “[play] a vital role as far as citizens’ use of e-government services is concerned” (Janssen et al. 2018, p. 663) needs to be supplemented by a far more fine-grained viewed on the types of public services under study and their unique riskiness.

Furthermore, with differentiating the causing entity and the perceiving entity, we are able to establish a relationship between these two parties that allows for the building of trust. Both trust and perceived risk necessitate some form of interaction between two entities. As our empirically grounded typology not only includes human entities as causes for risk perceptions but also systems or abstract technological infrastructures, we are able to open up the argumentative space created by Das and Teng (2004) towards non-human trustees and built the foundation for studying trust in e-government technologies.

Moreover, we conceptually disentangle risk perceptions and the resulting behaviour, risk-taking. Many of the articles in our sample treated perception and behaviour together, simply referring to both concepts as ‘perceived risks’. Despite this revelation, we decided to not include consequences of risk perceptions (trusting behaviour, use intention, usefulness) in our typology as theorists have repeatedly pointed to the need to separate the perception, which is a mental state, belief or expectation, and the behaviour resulting from this mental state (Das and Teng 2004; Mayer et al. 1995; Rotter 1971; Rousseau et al. 1998). In focussing our typology solely on risk perceptions, we provide a nuanced differentiation of risk perceptions and risk-taking in the e-government context.

## Conclusion

While risk and trust are “mirror images” (Das and Teng 2004), their use in contemporary e-government research stays often superficial. In this article, we set out to create a typology for trust-related e-government risks. With this typology, risks can be differentiated with regards to the source (i.e., where does the risk stem from), the perceiver (i.e., who perceives the risk), the type of risk, and the type of loss. This created typology allows a deeper understanding of different risks that users might perceive and, thus, also supports the creation of corresponding counter-measures to both minimize the objective risk and the risk perception. Overcoming the commonly used differentiation of trust in the Internet and trust in the government by providing a nuanced view on risk perceptions in e-government acceptance, we open the lid to the black-box ‘perceived risks’ and relate the different types of risk perceptions and risk sources back to trust and trusting behaviour. From this perspective, research needs consider where risks come from and what types of risks citizens perceive in order to better understand who citizens actually trust and why.

Coming back to the demonstration of the typology using a COVID-19 tracing app and the tax return service, we show that differentiating risks into the different types has inherent value in analysing behaviour of users. Building on such a nuanced view, different groups of users can be distinguished that perceive certain risks as more prevalent or important than other groups.

As such, our research has several implications for e-government practitioners. First and foremost, the typology can be used as an analytical framework to understand possible risks perceived by users of newly designed e-government services. Such an analysis can be one means to improve service acceptance. Secondly, our research also calls practitioners to evaluate the public trust into their suppliers. We could show that users might see the involvement of specific technology providers or system developers as being a situation of risk. As such, a trust-based analysis of suppliers and technologies used can prevent non-acceptance due to perceived risks.

The results of our research do not come without limitations. Firstly, the research is based on a qualitative analysis of published scholarly articles. As such, it might be limited due to a publication bias. However, situations where specific risks are “unpublishable” are hard to imagine. More importantly, our analysis is influenced by the individual analysis of the texts by the researchers. While we involved authors with different backgrounds and achieved a high degree of intercoder reliability, other authors might nevertheless derive a (slightly) different categorization of risks. Thus, future research needs to test and refine this typology. This is even more important considering the pace and scope of digital transformation as witnessed in public organisations right now, which requires a constant challenging of the typology and subsequent refinement. Secondly, our research is focused on e-government services for citizens. We searched for acceptance-related literature and consequently have a bias towards the citizens’ perception. This bias results in less risks for the administrations’ perspective; yet, many risks not listed in our overview may not be related to/require the employees’/organizations’ trust. We have excluded this perspective consciously. As such, our results should not be transferred to government-internal e-government without adaptation. Additionally, other researchers indicate that trust of the public administration in citizens and the society at large is also an important factor in e-government deployment and public administration actions in general (Bouckaert 2012)—a perspective that is not reflected in our typology. Here, future research needs to further investigate the complex interplay and reciprocal effects between actions of the public administration and its employees on the one hand and citizens and society on the other hand. Moreover, authors should be careful when transferring our results to government-to-business e-government services or to business-to-citizen services, e.g., e-business—a matter that is discussed in more detail below.

We see two main areas for future research based on our results. First, scholars should use and test the typology. Future research on trust and technology acceptance in the IS field should focus more often on the types of risks perceived by its users to enable a better understanding of what is actually inhibiting the acceptance of a system by its users. A corresponding research question to be answered could be “What configuration (or combination) of perceived risks inhibits the adoption and use of a system?”. To this end, researchers could collect primary data from end users (citizens) on their risk perceptions either through qualitative interviews or through quantitative survey research. Second, scholars could work on transferring the typology to other sectors (e-business, IS in general). A potential research question could be “How can risks perceived in the context of information systems in general be typologised?”. One could argue that the typology we have developed is domain-agnostic: In the private sector, risks can stem from service providers, technology, developers, third parties and deal with quality, confidentiality, or integrity of information and data. Also, corresponding losses can be of different types (with maybe less emphasis on the perceived risk to lose democratic rights). However, as our research was purely focused on e-government, this transfer and generalization should be subject to future research. Next to these two main areas, our research shows that the terms ‘risk’ and ‘perceived risk’ are often used without proper definition in previous research. Thus, we call future research to properly define the used constructs to prevent misunderstandings and misinterpretations.

## Footnotes

1 The full search string in both databases was (‘e-government’ OR ‘e-gov’ OR ‘electronic government’ OR ‘e-government’ OR ‘online government’ OR ‘digital government’ OR ‘public online service’ OR ‘online government’ OR ‘digital public service’) AND (‘trust’ OR ‘distrust’ OR ‘mistrust’) AND (‘adoption’ OR ‘acceptance’ OR ‘intention’ OR ‘use’ OR ‘usage’ OR ‘rejection’ OR ‘resistance’ OR ‘non-adoption’). Please note that there was no restriction with regards to publication date. Since the DGRL includes only references on digital government or electronic government, this library was searched using only the trust- and adoption-related keywords.

2 The first three authors conducted the data analysis.

3 In the corresponding verdict, the court argued in favor of the government.
